# Using Technology to Connect the Generations and Understand Lifelong Learning

**DOI:** 10.1093/geroni/igaa057.1816

**Published:** 2020-12-16

**Authors:** Laura Donorfio, Brian Chapman

**Affiliations:** University of Connecticut, Waterbury, Connecticut, United States

## Abstract

The University of Connecticut (UConn) has a thriving Osher Lifelong Learning Institute (OLLI), which has existed for over a decade on one of their regional campuses (700+ members). Intergenerational classes are utilized, but connecting UConn students with OLLI members outside of these classes in an effective, meaningful way is a challenge. A successful model developed within an adulthood and aging class to connect the generations outside of the classroom utilizes technology as a bridge. The two most successful activities will be highlighted. The first is a “technology clinic,” which requires students to pair up with OLLI members to assist them with technological needs. The second requires students to create a podcast by interviewing an OLLI member on the importance of lifelong learning, which is uploaded to the campus OLLI website. Implications for both generations, bi-directional affective change, and inclusivity of older learners in the classroom and beyond will be discussed.

